# Using environment-sensitive tetramethylated thiophene-BODIPY fluorophores in DNA probes for studying effector-induced conformational changes of protein–DNA complexes†

**DOI:** 10.1039/d4cb00260a

**Published:** 2025-01-02

**Authors:** Markéta Šoltysová, Pedro Güixens-Gallardo, Irena Sieglová, Anna Soldánová, Veronika Krejčiříková, Milan Fábry, Jiří Brynda, Petro Khoroshyy, Michal Hocek, Pavlína Řezáčová

**Affiliations:** a Institute of Organic Chemistry and Biochemistry, Czech Academy of Sciences Flemingovo n. 2 Prague 6 Czechia hocek@uochb.cas.cz rezacova@uochb.cas.cz; b Department of Organic Chemistry, Faculty of Science, Charles University Hlavova 8 CZ-12843 Prague 2 Czechia

## Abstract

The LutR protein represses the transcription of genes encoding enzymes for the utilization of l-lactate in *Bacillus subtilis* through binding to a specific DNA region. In this study, we employed oligonucleotide probes modified by viscosity-sensitive tetramethylated thiophene-BODIPY fluorophores to investigate the impact of selected metabolites on the LutR–DNA complex. Our goal was to identify the effector molecule whose binding alters the protein–DNA affinity, thereby enabling gene transcription. The designed DNA probes exhibited distinctive responses to the binding and release of the protein, characterized by significant alterations in fluorescence lifetime. Through this method, we have identified l-lactate as the sole metabolite exerting a substantial modulating effect on the protein–DNA interaction and thus confirmed its role as an effector molecule. Moreover, we showed that our approach was able to follow conformation changes affecting affinity, which were not captured by other methods commonly used to study the protein–DNA interaction, such as electro-mobility shift assays and florescence anisotropy binding studies. This work underlines the potential of environment-sensitive fluorophore-linked nucleotide modifications, *i.e.* dC^TBdp^, for studying the dynamics and subtle changes of protein–DNA interactions.

## Abstract 2

Using fluorophore-linked probes, we revealed conformational changes affecting protein–DNA affinity that were undetectable using traditional methods. We identified l-lactate as the effector molecule of transcriptional repressor LutR in *B. subtilis*.

## Graphical abstract

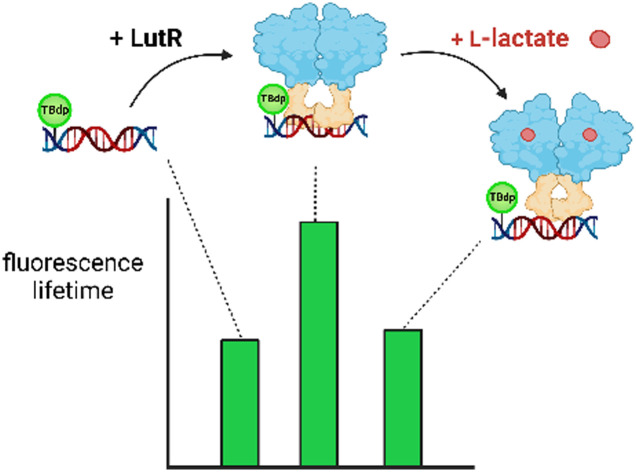

## Introduction

Fluorescent nucleotides are widely used for the labeling of nucleic acids, enabling their imaging and quantification.^1^ Diverse fluorophore-linked 2′-deoxyribonucleotide triphosphates (dN^Fl^TPs) have been utilized in the enzymatic synthesis of labeled DNA using DNA polymerases through primer extension.^2^ Modification with environment-sensitive fluorophores^3^ is particularly useful, as it can be used for studying hybridization,^4–7^ secondary structure changes,^8–10^ nascent DNA synthesis^11^ or protein–DNA interactions.^12–17^ Recently, viscosity-sensitive molecular rotors have frequently been used for studying biomolecular interactions, which result in hindering the molecular rotation and thus changes in the fluorescence lifetime.^18,19^ In nucleotide and nucleic acids,^20^ diverse modified fluorescent molecular rotors based on hetaryl-nucleobases,^21^ GFP-like fluorophores,^22,23^ stilbene and analogues^16,17^ or substituted *meso*-phenyl boron dipyrromethene (BODIPY) fluorophores^13–15^ that respond to protein–DNA binding by changing the fluorescence intensity and/or lifetime have been reported. However, actual applications of fluorophore-labeled DNA probes for studying biological questions are scarce. In the study presented here, we focused on the previously reported modified nucleoside triphosphate dC^TBdp^TP,^13^ containing a tetramethylated thiophene-BODIPY moiety (TBdp), as a building block for the construction of DNA probes for studying the binding mode of a transcriptional regulator.

Metabolic transcriptional repressors are proteins that act as molecular switches controlling the transcription of specific genes involved in bacterial metabolism.^24^ These repressors typically possess two domains: an N-terminal DNA-binding domain (DBD) and a C-terminal effector-binding domain (EBD).^25^ The DBD recognizes a specific DNA sequence called a DNA operator, which is often located near promoter regions of regulated genes. When an effector molecule binds to an EBD, it induces a conformational change in the protein, reducing its affinity for the DNA operator and leading to the release of the protein from the DNA. Consequently, the transcription of the regulated genes is activated.^26^

Functional studies of bacterial metabolic repressors are crucial for unraveling underlying regulatory mechanisms, discovering novel metabolic pathways, optimizing industrial processes, and developing strategies to combat microbial pathogens. A thorough understanding of how proteins interact with DNA and how this interaction is influenced by small molecules represents pivotal stages in these functional investigations. Consequently, the development of innovative approaches, like the sensitive probes introduced here, holds significant importance.

In this study, we investigated the interaction between a specific bacterial repressor, l-lactate utilization repressor (LutR) from *Bacillus subtilis*, and its DNA operator modified by the dC^TBdp^ fluorophore attached at specific positions.^12^ Additionally, we used this probe to identify a molecule that acts as an effector of LutR.

LutR, initially named YvfI,^27^ belongs to the GntR family, which is one of the largest families of bacterial transcriptional regulators.^28^ It consists of 240 amino-acid residues and forms two distinct domains: a 78 residue long N-terminal DBD and a 162-residue C-terminal EBD. LutR plays a regulatory role in the utilization of l-lactate in undomesticated *B. subtilis* strains.^29,30^ Specifically, it acts as a repressor of the *lutABC* operon, which codes for three iron–sulfur proteins essential for l-lactate utilization, as well as the *lutP* gene, encoding l-lactate permease. The binding site for LutR, characterized by the consensus inverted repeat sequence TCATC-N1-GATGA, is located downstream of the *lutA* or *lutP* promoters.^30^ Previous studies have shown that the transcription of both *lutABC* and *lutP* genes is induced in a dose-dependent manner by l-lactate *in vivo*, but the effect of l-lactate and its metabolites on the interaction between LutR and the DNA operator was never demonstrated using an electrophoretic mobility shift assay (EMSA).^30^

It is noteworthy that a shorter LutR protein variant exists in laboratory strains of *B. subtilis*. This variant, resulting from a mutation that eliminates the first 21 amino acids, experiences altered DNA recognition specificity and functions as a global regulator, influencing genes associated with the transition from the exponential growth phase to the stationary phase of bacterial populations.^30,31^ To prevent confusion, our study specifically targeted the full-length LutR variant identified in the undomesticated strain RO-NN-1. In this strain, the full-length LutR variant controls the transcription of genes essential for l-lactate utilization. The role of LutR in the regulation of lactate transport was recently confirmed for *Bacillus coagulans* DSM1.^32^

We envisaged dC^TBdp^-modified oligonucleotide probes as tools for studying the interaction between LutR and DNA in the presence of various metabolites using fluorescence-based techniques. We selected this approach to uncover which of these molecules function as effectors and to explore the potential of dC^TBdp^-modified oligonucleotides in studying protein–DNA interactions.

## Results

### Design of modified oligonucleotides based on a protein–DNA structural model

We started this project by designing and constructing a structural model where the DNA-binding domain of LutR (LutR–DBD) is bound to its DNA operator. The model allowed us to verify that none of our oligo-modifications would severely hinder the interaction between LutR and DNA. We determined an X-ray structure of the free LutR–DBD (residues 2–78) by molecular replacement and refined the structure to the resolution of 1.46 Å (Table S1, ESI†). The crystal belonged to the *P*222 space group and contained 30.8% of solvent. The asymmetric unit comprised one protein molecule, all residues of which were modeled into a well-defined electron density map, with the exception of the first six N-terminal residues representing a cloning artifact and the first native residue of the LutR–DBD. The non-protein electron density map was explained by 81 water molecules and two sodium ions.

**Fig. 1:**
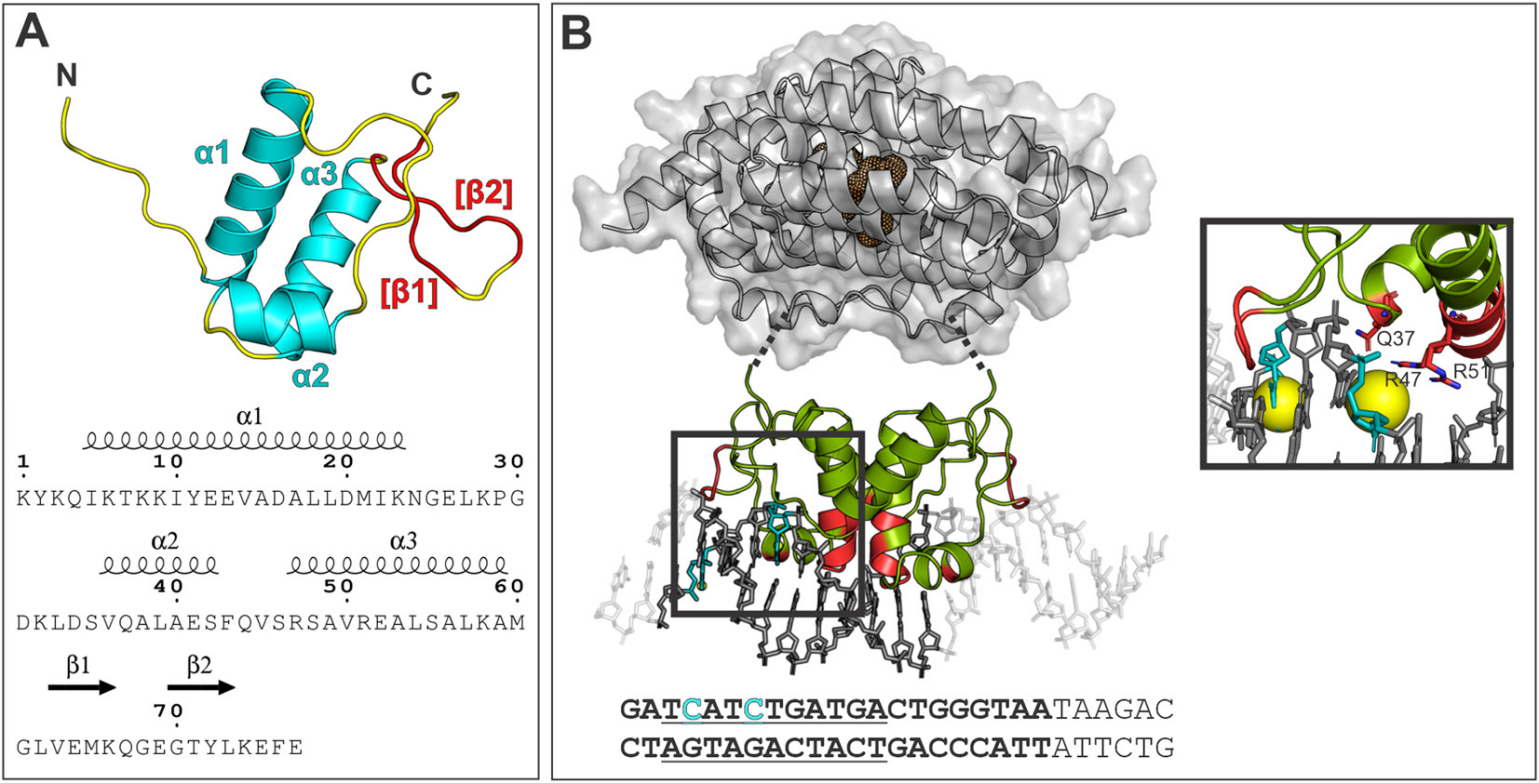
(A) Cartoon representation of the LutR–DBD crystal structure. Secondary structures are distinguished by color and labeled as follows: α helices in cyan, disordered regions (loops) in yellow and the assumed β-sheet region (wing) in red. At the bottom, the secondary structure prediction is shown. (B) Model of the LutR–DBD dimer bound to the modified DNA. The hypothetical position of EBDs is shown in gray, the linkers between the DBDs and EBDs are represented by dashed lines, and the effector-binding site is indicated as a dark mesh sphere. Protein areas involved in the interaction with DNA are highlighted in red. The sequence of the labeled DNA strand is shown below. The dC^TBdp^ bases are shown in cyan in the 3D model as well as the sequence. The TBdp modifications are shown as yellow spheres in the zoomed-in box. The 21-bp DNA duplex used for the modeling is in bold, and the operator sequence is underlined.

The LutR–DBD structure consists of one of the most abundant DNA-binding motifs, winged helix–turn–helix (wHTH) (Fig. 1A), structurally homologous to transcriptional regulators of the MarR family.^33,34^ The topology of the LutR–DBD starts with an unstructured N-terminus followed by three α-helices: α1 (residues 10–25), α2 (residues 36–43) and α3 (residues 53–61), which are connected by loops. The expected β-strands folded into the wing were not revealed in our DNA-free structure. However, it is highly plausible that they are formed upon DNA binding, and their positions were predicted by the servers, AlphaFold 2^35^ and JPred4^36^ for amino acid regions 63–66 and 69–73 (Fig. 1A).

The crystal structure revealed a LutR–DBD monomer. However, the formation of a dimer both in the presence and absence of DNA was confirmed by size exclusion chromatography (SEC) (Fig. S1A, ESI†). The dimer represents the biological unit that binds to the DNA operator, as observed also for other structural homologs such as the arabinose repressor from *B. subtilis* (AraR).

The structure of the AraR–DBD bound to DNA was previously determined for several DNA sequences,^37^ and we used one of the experimental structures, deposited in the PDB database under code 4EGY, to compose a model of the LutR–DBD bound to a DNA duplex containing the known operator sequence 5′-TCATCTGATGA-3′ (Fig. 1A). We assume that the LutR–DBD binds as a dimer into a major groove by the recognition helix α3 and that this interaction is further supported by wings that wedge into the adjacent minor grooves (Fig. 1B). We predicted the amino-acid residues that might be involved in the interaction with DNA (Fig. 1B) based on the protein-binding sequence signatures of LutR homologs analyzed by the 3D-footprint server.^38^ Finally, we used the structural model to propose suitable sites for modification. In particular, we selected two C bases in the DNA sequence. The first modified base, C27, is located upstream of the operator sequence and is expected to be involved in non-specific interactions with the wing region of LutR. The second base, C24, is located in the major groove where the interaction with the recognition α3 helix of LutR is expected (Fig. 1B).

### Preparation of modified DNA probes

**Fig. 2:**
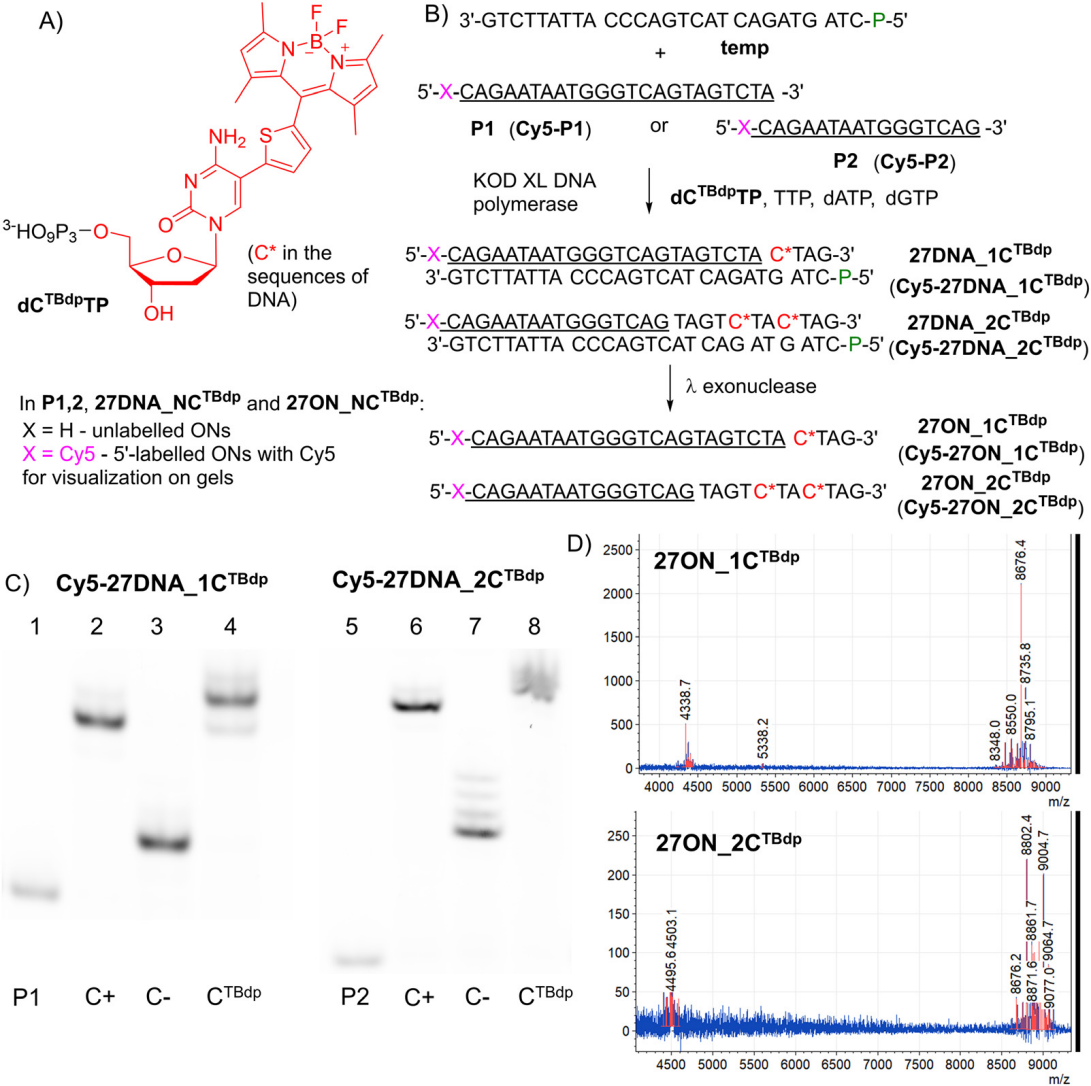
(A) Structural formula of TBdp-modified deoxycytidine monophosphate. (B) PEX experiment in the presence of KOD XL DNA polymerase using Cy5 fluorescently labeled (pink X) primers (P1, P2), resulting in singly or doubly TBdp-modified 27 bp long DNA duplexes (27DNA_1C^TBdp^ and 27DNA_2C^TBdp^) and single-strand oligonucleotides (27ON_1C^TBdp^ and 27ON_2C^TBdp^). The modified C base is highlighted in red and labeled with an asterisk. (C) EMSA experiment: Cy5-P1 (lanes 1–4) or Cy5-P2 (lanes 5–8). “P1” and “P2” indicate primers shown in panel B, “C+” labels the PEX products upon the use of natural dNTPs, “C−” labels the PEX products without the addition of dCTPs, “C^TBdp^” labels the PEX products upon the use of modified dC^TBdp^TPs and three corresponding natural dNTPs. (D) MALDI mass spectra showing the full range of modified oligonucleotides (ONs) using non-Cy5-labeled primers. The upper graph is for the singly modified ON (8676.4) and the lower one is for the doubly modified ON (9004.7).

The designed duplex DNA probes were synthesized enzymatically through primer extension (PEX) using dC^TBdp^TP^13^ in combination with three other natural dNTPs (Fig. 2B), utilizing a template with a 5′-end phosphorylation that does not impact the protein–DNA interaction and allows its digestion by lambda exonuclease. We employed two primer sequences: a 14-mer and a 21-mer, both complementary to the same template (Table S2, ESI†). These primers permitted the incorporation of either one dC^TBdp^ modification at position 7 (the resulting duplex is denoted 27DNA_1C^TBdp^) or the simultaneous incorporation of two modifications at positions 4 and 7 (the resulting duplex is denoted 27DNA_2C^TBdp^) during the PEX process with KOD XL DNA polymerase.

To monitor the PEX reaction and optimize the conditions for the full extension, we utilized Cy5-labeled primers. Additionally, these labeled primers enabled the generation of DNA duplexes for subsequent EMSA experiments assessing the binding capabilities of LutR–DNA. For time-correlated single photon counting (TCSPC) experiments, non-labeled primers were used, resulting in dC^TBdp^-containing DNA duplexes without the 5′-Cy5 labels. An aliquot of digested DNA duplexes was subjected to MALDI mass spectrometry analysis to assure the proper incorporation of the modified dC^TBdp^ nucleotide(s) (Fig. 2D).

**Fig. 3:**
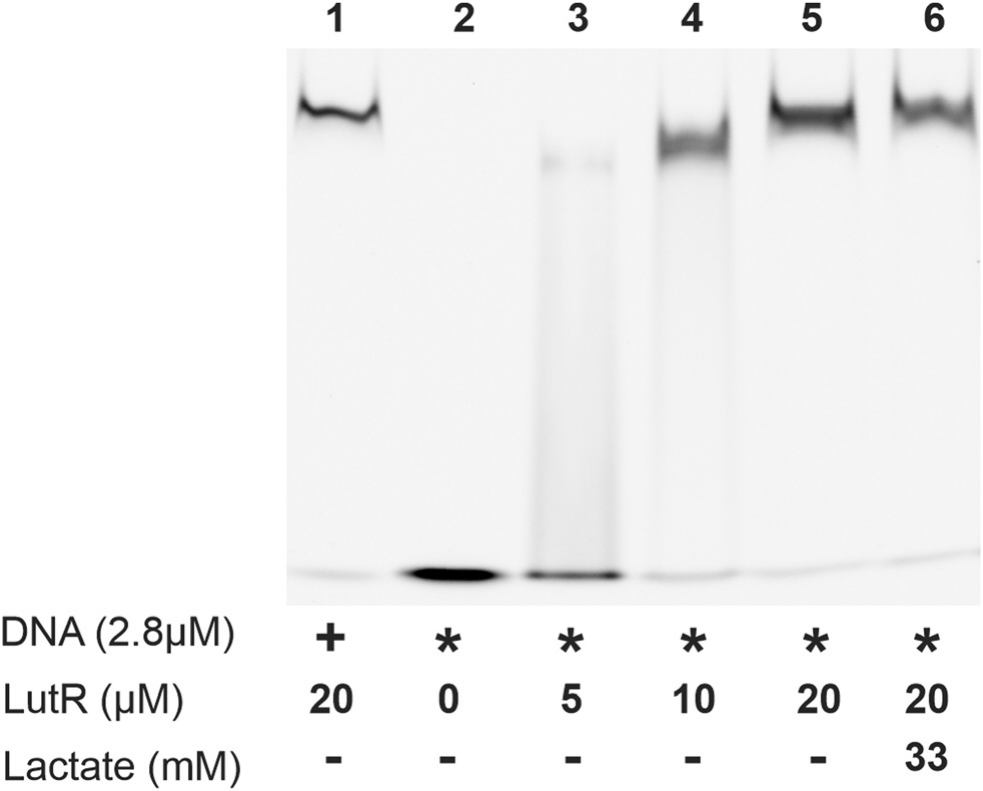
EMSA experiment showing the interaction of Cy5-labeled DNA and LutR with (+) or without (−) the addition of sodium l-lactate. Plus symbols (+) indicate the use of the native DNA duplex; asterisks (*) indicate the use of 27DNA_2C^TBdp^.

The binding affinity of LutR towards the modified duplexes was examined using an EMSA (Fig. 3). The results show that LutR at a concentration of 10 μM could completely retard the DNA containing the operator sequence. A similar observation was made in EMSA experiments performed in the presence of increasing amounts of LutR–DBD and unmodified DNA (Fig. S2, ESI†) where a dose-dependent shift was observed for cognate 15-bp DNA (Fig. S3, ESI†). These findings experimentally confirm our structural model assumptions, demonstrating that modifications of the two bases do not hinder the interaction.

Furthermore, we tested the effect of l-lactate and found that the addition of this metabolite at a concentration of 33 mM does not inhibit the binding of LutR to its DNA operator in an EMSA assay. A similar result was reported by Chiu *et al.*, who conducted an EMSA experiment probing l-lactate along with several other metabolites and found no effect of any of them.^30^

### Using dC^TBdp^-modified DNA and TCSPC detection to identify the effectors of LutR

Previous studies have shown that l-lactate inhibits the binding of LutR to its operator *in vivo*. However, no effect has been proven *in vitro* using an EMSA,^30^ be it for l-lactate or any of the other related metabolites, which had also been suspected of acting as effector molecules. For this reason, we tested all the candidate molecules – l-lactate, l-alanine, pyruvate, acetate and acetyl-CoA (Fig. S4, ESI†).

**Fig. 4:**
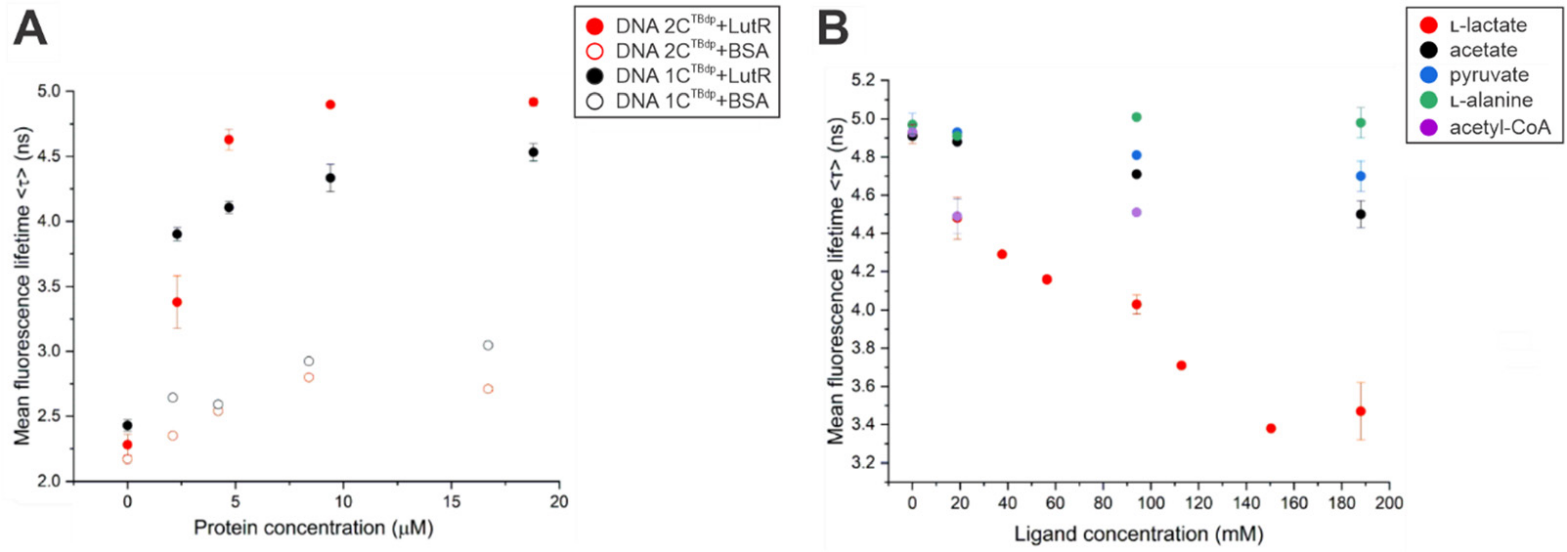
Confirmation of the binding of LutR to singly and doubly modified DNA and analysis of the potential effector activity of metabolites suspected of acting as effectors. (A) Titration of 27DNA_2C^TBdp^ (red) and 27DNA_1C^TBdp^ (black) at a constant concentration of 0.2 μM with LutR (full circle) and BSA (open circle) proteins. (B) Titration of the LutR–27DNA_2C^TBdp^ complex with the tested metabolites l-lactate (red), sodium acetate (black), sodium pyruvate (blue), l-alanine (green) and acetyl-CoA (purple). The final concentrations of 27DNA_2C^TBdp^ and LutR in the reaction mix were 1.35 μM and 3.2 μM, respectively.

In this study, we closely monitored the molecular interactions of the LutR–DNA complex with l-lactate and other potential effector molecules using the TCSPC method. The dC^TBdp^ nucleotide is a fluorescent rotor known for its sensitivity to microenvironment viscosity, and once incorporated into DNA, it acts as a TCSPC probe. When bound to a protein, dC^TBdp^-containing DNA exhibits a longer fluorescence lifetime due to the restricted rotation of the fluorescent rotor.^13^ Addition of increasing amounts of LutR protein to DNA resulted in increased mean fluorescence lifetime, suggesting protein binding and reaching plateau at a concentration around 10 μM (Fig. 4A). Our DNA duplexes 27DNA_1C^TBdp^ and 27DNA_2C^TBdp^ increased the mean fluorescence lifetime by up to 210% upon the addition of the LutR protein. The negative control experiment with bovine serum albumin (BSA) only increased the signal by 20% with respect to the free DNA (Fig. 4A, values in Table S3, ESI†).

The effects of the potential effector molecules on protein–DNA binding were measured with 27DNA_2C^TBdp^ because it displayed a stronger response upon the addition of LutR. Addition of increasing amounts of l-lactate resulted in a decrease of mean florescence lifetime, while no such trend was observed for other tested metabolites (Fig. 4B).

Due to limitations in the amount of modified DNA available, we triplicated values of 1, 5, and 10, while the remaining values (2, 3, 6, and 8) represented an additive amount of modulator, diluting the protein solution. Although there are some outliers, the dose-dependent trend observed was clear: a decrease in lifetime with the addition of l-lactate, reaching a minimum (65% signal reduction) at eight molar equivalents. Other metabolites, by contrast, produced changes of less than 10% (see Fig. 4B).

### Binding studies using the commonly used fluorescein probe

**Fig. 5:**
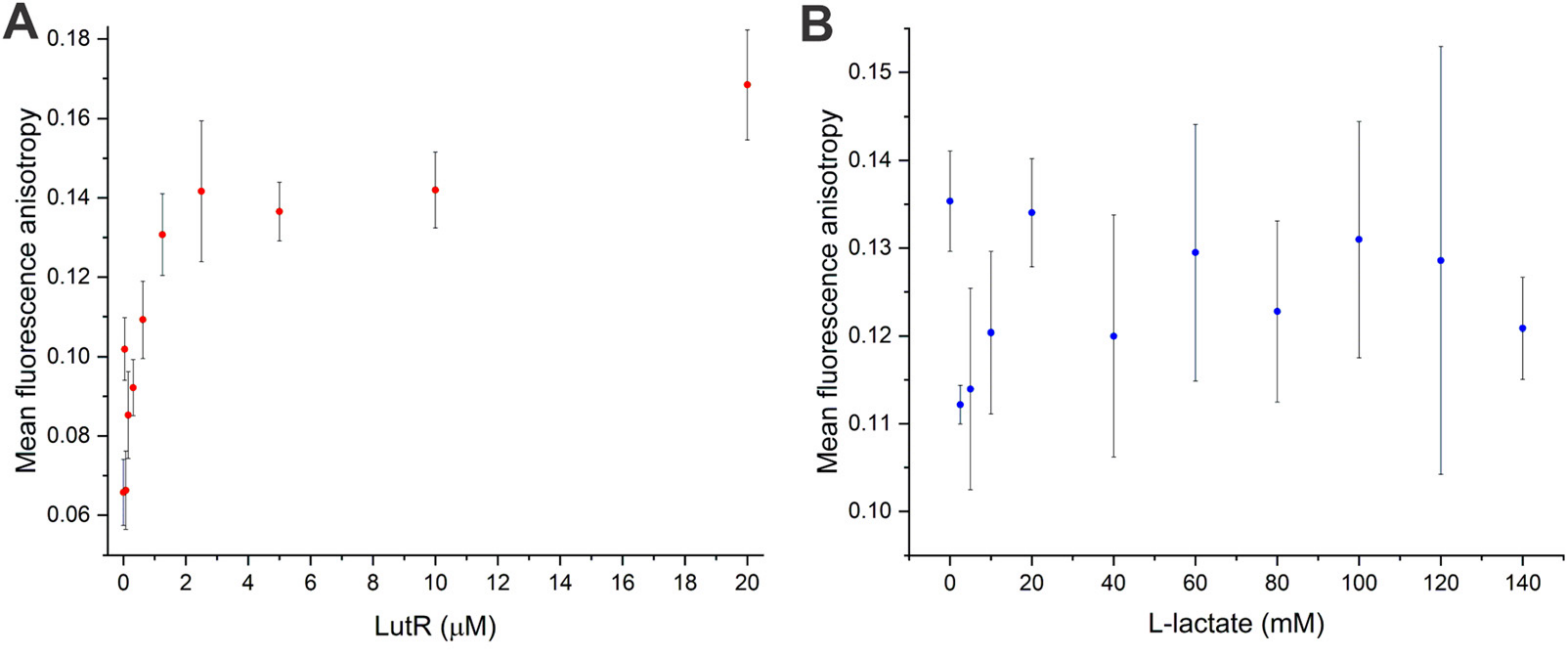
Fluorescence anisotropy binding studies. (A) Titration of 6FAM-27DNA (5 nM) with the increasing concentration of LutR. (B) Titration of the mixture of LutR (10 μM) and 6FAM-27DNA (5 nM) with the increasing concentration of l-lactate. All data points were measured in duplicates or triplicates.

To complement the TCSPC observations and compare the sensitivity of TBdp modification with commonly used probes, we employed an orthogonal fluorescence-based method: fluorescence anisotropy measurement using the 5′ end 6FAM labeled 27DNA duplex. While the anisotropy signal increased with protein concentration (Fig. 5A) indicating the LutR–DNA interaction, no decrease was observed upon the addition of l-lactate (Fig. 5B). These results indicate that the addition of l-lactate does not cause full dissociation of the protein from DNA.

### Oligomeric states of LutR and its complexes

To determine whether ligand binding affects oligomerization or hydrodynamic properties of the LutR DNA complex, we conducted size exclusion chromatography (SEC) analysis (Fig. S5, ESI†). The elution profiles revealed a shift in the protein signal upon DNA binding, indicating a dimer bound to a duplex. However, no change in the LutR DNA signal was detected in the presence of 50 mM l-lactate.

### LutR binds l-lactate

**Fig. 6:**
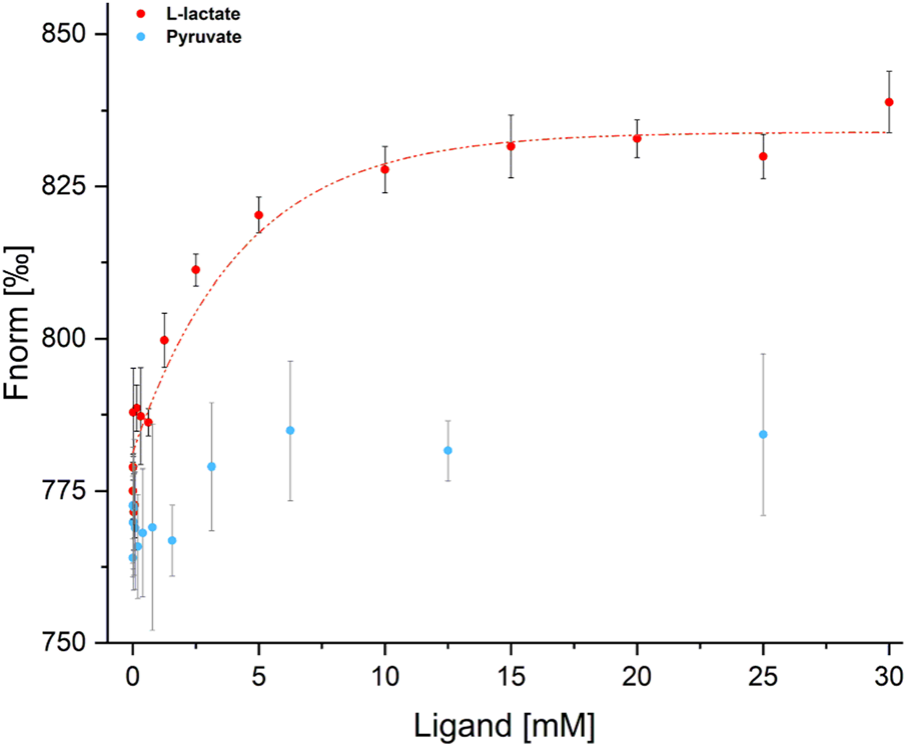
MST analysis of LutR interaction with l-lactate (red) and pyruvate (blue). Data from the LutR–l-lactate measurement were used to derive the saturation curve (red line) and *K*_d_ value (2.3 ± 0.005 mM). Measurements were carried out in triplicates.

To verify LutR affinity for l-lactate, we examined the binding affinity of l-lactate to the free LutR protein using microscale thermophoresis (MST). This experiment analyzed the protein behavior in a temperature gradient (Fig. 6) in both the absence and presence of l-lactate and pyruvate. The results clearly demonstrate an interaction with l-lactate with the calculated *K*_d_ value of 2.3 ± 0.005 mM. On the other hand, the change in fluorescence signal in the presence of pyruvate is negligible and does not indicate ligand binding.

## Discussion

We utilized synthetic DNA probes containing cytosines modified by a viscosity-sensitive tetramethylated thiophene BODIPY fluorophore (dC^TBdp^) to follow how a protein interacts with DNA. We showed that our novel TBdp modification represents an excellent alternative to commonly used end-labelling probes, which are not sensitive to subtle environmental changes.

We focused on the transcriptional repressor LutR from *B. subtilis* and the corresponding DNA duplex matching its DNA operator sequence. Through the incorporation of this modification and the application of the time-correlated single photon counting (TCSPC) technique, we identified the actual effector molecule that regulates the affinity of LutR towards DNA. Our finding of this activity corroborates the results of earlier *in vivo* studies suggesting l-lactate as an effector even though its effect on protein–DNA interactions had previously never been confirmed *in vitro*.^30^

Using the known sequence of the LutR operator, we selected two positions where cytosines could be modified. A model constructed using the newly determined crystal structure of the LutR–DBD (Fig. 1) guided the selection of bases C21 and C24 for TBdp modification and allowed us to predict the surrounding amino-acid residues and anticipate their interactions or steric clashes. While the modification at C21 was not expected to pose any problems, as it is not involved in any crucial protein contact and is located upstream of the operator sequence, the modification at C24 is situated in the DNA major groove, where the interaction with the α3 recognition helix is anticipated. Contrary to expectations, both TBdp locations were shown to be compatible with LutR–DNA complex formation in EMSA experiments (Fig. 3) and produced signals in TCSPC analysis (Fig. 4).

The dC^TBdp^ modification used as a TCSPC probe generated an exceptional difference in mean fluorescence lifetime^39^ (of up to about 2.7 ns) between the free DNA duplex and the protein–DNA complex (Table S3, ESI†). Such well-defined DNA states allowed us to monitor the dissociation of the LutR–DNA complex upon the addition of small molecules that were suspected of acting as effectors.

Unlike EMSA experiments conducted by us and others,^40^ TCSPC clearly demonstrated that l-lactate has a negative effect on the LutR–DNA interaction and we see a dose-dependent trend (Fig. 4A). Additionally, we determined the l-lactate affinity to LutR with the *K*_d_ value of 2.3 mM. This value lies within the range of intracellular concentrations for this metabolite^41^ but is unexpectedly high for an effector molecule. We suggest that this low affinity could result from the absence of SinR, a major transcriptional regulator of biofilm formation that works cooperatively with LutR to repress the *lutABC* operon. The activity of LutR is closely tied to SinR, ensuring coordinated regulation of l-lactate utilization and biofilm formation.^29^ Therefore, it is plausible that SinR interaction *in vivo* influences LutR affinity for l-lactate.

The contradictory results of EMSA and fluorescence anisotropy binding studies infer that the binding of the effector does not lead to a large change in the protein–DNA complex radius or cause complete dissociation.

Such behavior was observed in other transcriptional repressors, *e.g.*, the extensively studied central glycolytic regulator (CggR) in *B. subtilis*. The dissociation of CggR from DNA was not observed in the EMSA upon the addition of the confirmed effector, fructose-1,6-bis-phosphate (FBP). However, other biophysical and structural analyses provided clear evidence that FBP induces subtle structural changes in EBDs that consequently impact the affinity of CggR to DNA.^42–45^ Such a loose complex allows the RNA-polymerase to read through the DNA operator and continue with the gene transcription.^46^ A similar scenario might be anticipated for LutR.

**Fig. 7:**
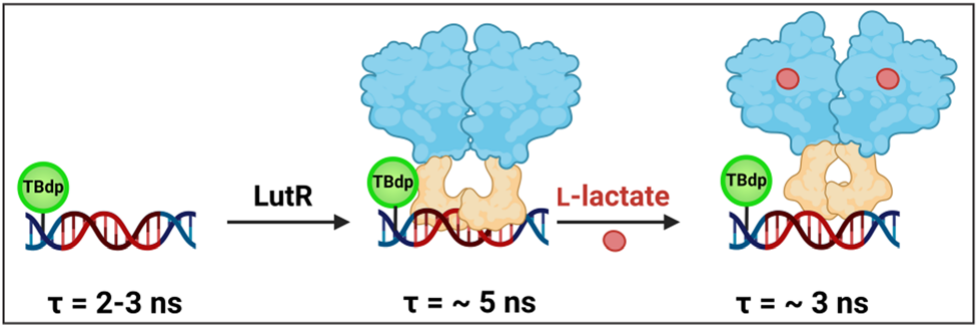
Schematic representation of the interaction between the LutR protein and fluorescent DNA probes. Conformational changes induced by lactate binding, leading to changes in fluorescence lifetime of the dC^TBdp^-modified DNA probe and their effect on mean fluorescence lifetime (*τ*) are indicated. The effector-binding domain and DNA-binding domain are colored blue and orange, respectively.

Although the precise structural basis for the conformational changes induced by the effector remains to be elucidated, we constructed a schematic model explaining our observations (Fig. 7). The two-domain LutR protein binds to the DNA operator as a dimer, restricting the rotation of the TBdp probe, as reflected by an increase in the mean fluorescence lifetime. When l-lactate binds to the EBD, it induces a conformational change that is transmitted to the DBD, affecting the protein–DNA interface, while LutR remains attached to the DNA. This change creates room for the TBdp fluorescent probe to rotate, leading to a substantial drop in the mean fluorescence lifetime (Fig. 7).

We might conclude that dC^TBdp^-modified DNA probes are valuable for studying protein–DNA interactions, particularly when induced changes do not significantly alter the overall structure of the complex or lead to its dissociation. Positioning the TBdp-modified nucleotide next to the DNA recognition motif did not hinder protein interaction but provided the advantage of detecting subtle conformational changes. These changes are often undetectable using conventional methods such as EMSA or fluorescence anisotropy experiments with commonly used fluorescein-labeled DNA. By using the dC^TBdp^-modified DNA probes, we identified l-lactate as the sole effector molecule of LutR, whose binding allows the transcription of the *lutABC* operon.^30^ Moreover, our study proved that DNA probes containing environment-sensitive fluorophores represent a potent tool for studying protein–DNA interactions including even minor conformational changes undetectable by other methods and suggest potential for the high-throughput screening of small molecules affecting protein–DNA interactions using commercial TCSPC plate readers.^47^

## Materials and methods

### Preparation of full-length LutR and LutR–DBD proteins

Full-length LutR (LutR-FL, amino-acid residues 2–240, NCBI sequence NP_391298.2) and LutR–DBD (amino-acid residues 2–78) were cloned into the multicopy expression plasmid derived from the pMCSG7 vector originally designed for ligase-free cloning.^48^ This plasmid fused the proteins with N-terminal hexahistidine (His_6_) tags and the tobacco etch virus protease (TEV PR) recognition sites. Upon the TEV PR cleavage of the His_6_ tags, the five amino-acid sequences (SNAAS) remained at the N-termini of the protein products as cloning artifacts.

Both proteins were overexpressed in *E. coli* BL21 (DE3) cells grown on Luria-Bertani broth (Sigma-Aldrich, USA) supplemented with 0.4% (v/v) glycerol and 100 μg ml^−1^ ampicillin. The bacteria were grown at 37 °C until the value of OD_550nm_ reached about 1. Afterwards, the cultures were transferred to 20 °C, and the gene expression was induced with 100 μM (LutR–DBD) or 250 μM (LutR–FL) β-d-1-thiogalactopyranoside and cultivated for an additional 14 h. Bacterial cells were harvested by centrifugation (3300*g* at 4 °C for 20 min) and resuspended in lysis buffers [LutR–DBD: 50 mM Tris·HCl, pH 7.5, 500 mM NaCl, 5 mM imidazole, 5% (v/v) glycerol, and the recommended amount of protease inhibitor cocktail tablets cOmplete™ (Roche, Switzerland); LutR–FL: the same buffer composition with the addition of 5 mM β-mercaptoethanol] in a volume of 10 ml per gram of cells. The suspensions were further sonicated and centrifuged (25 000*g* at 4 °C for 30 min). The soluble fractions of the lysates were further subjected to nickel chelation chromatography on a His-select nickel affinity gel (Ni–NTA) column (Sigma-Aldrich, USA) equilibrated in loading buffers, which were of the same compositions as the lysis buffers but without the protease inhibitor. The remaining protein contamination and His_6_-tagged proteins were step-eluted with loading buffers supplemented with 30 mM and 250 mM imidazole, respectively. In the case of the LutR–DBD, the His_6_ tag was cleaved off by dialysis in the presence of TEV PR against a dialysis buffer [20 mM Tris·HCl, pH 7.5, 500 mM NaCl, 5% (v/v) glycerol] at room temperature for approximately 14 h. The cleaved His_6_ tag and TEV PR were removed by a second run of Ni–NTA chromatography. The purified LutR–DBD and His_6_-tagged LutR–FL solutions were dialyzed against a storage buffer of the identical composition (20 mM Tris·HCl, pH 7.5, 250 mM NaCl, 5 mM β-mercaptoethanol) and concentrated on Amicon Ultra concentrators (Millipore, USA). The concentrations were approximately 32 mg ml^−1^ for the LutR–DBD and 10 mg ml^−1^ for LutR–FL, as estimated by measuring absorption at 280 nm using theoretical absorption coefficients of 0.495 l g^−1^ cm^−1^ and 0.538 l g^−1^ cm^−1^ for the LutR–DBD and LutR–FL, respectively.

### Fluorescence anisotropy

Fluorescence anisotropy experiments were performed using a FluoroMax-4 fluorometer (Horiba Scientific, Japan). 100-μl samples were measured in a quartz cuvette. The fluorescence signal was measured using a constant concentration (5 nM) of DNA labelled with 6-carboxyfluorescein (6FAM) at the 5′ end, excited with light at a wavelength of 492 nm. The fluorescence spectra at different positions of the polarizers were recorded in the wavelength range of 505–600 nm with 5 nm steps. For both excitation and emission, the slit width was set to 5 nm. For single measurement, anisotropy data points measured at 515, 520 and 525 nm were averaged. All samples were measured in duplicates or triplicates.

The mixtures of LutR and DNA were incubated for at least 30 min on ice before the measurement. In the case of titration experiment with l-lactate, the protein and DNA were first incubated and subsequently diluted with l-lactate and the protein storage buffer (20 mM Tris·HCl, 250 mM NaCl, 0.02% (v/v) β-mercaptoethanol) up to final concentrations of 10 μM and 5 nM, respectively.

### Analytical size-exclusion chromatography (SEC)

Analytical SEC experiments were performed on an FPLC Äkta Basic machine (Amersham Biosciences, Great Britain) and a Superdex 200 10/300 GL Tricorn column at room temperature. The elution profiles were monitored online at wavelengths of 280, 254 or 260, and 220 nm. The column was calibrated using Superdex 200 molecular-weight (MW) standards in PBS buffer (137 mM NaCl, 10 mM Na_2_HPO_4_, 1.8 mM KH_2_PO_4_m 2.7 mM KCl, pH 7.4). The MW standards were: thyroglobulin (670 000 Da), aldolase (158 000 Da), conalbumin (75 000 Da), carbonic anhydrase (29 000 Da), and ribonuclease A (13 700 Da)] or [thyroglobulin (670 000 Da), γ-globulin (158 000 Da), ovalbumin (44 000 Da), myoglobulin (17 000 Da), and vitamin B12 (1350 Da). The void volume was determined based on blue dextran.

LutR–DBD analyses were carried out in the PBS buffer as well. The protein concentration was always diluted to 3 mg ml^−1^ (33.2 μM). The protein–DNA complex was mixed in a molar ratio of 2 : 1. The sample volume loaded onto the column was 100 μl in all instances.

The full-length LutR analyses were performed in the storage buffer (20 mM Tris·HCl, 250 mM NaCl, 0.02% (v/v) β-mercaptoethanol, pH 7.5). The protein concentration was always diluted to 2 mg ml^−1^ (66.7 μM) and the DNA concentration was diluted to 50 μM. The protein–DNA complex was mixed in a molar ratio of 2 : 1.5. The concentration of l-lactate in the mixture was 50 mM. The sample volume loaded onto the column was 100 μl in all instances.

### Microscale thermophoresis (MST)

MST experiments were performed in a Monolith NT.LabelFree MST instrument (Nanotemper Technologies) using Monolith NT.LabelFree capillaries. The LED (excitation) power and IR-laser (MST) power were set at 15% and 20%, respectively. Data were analyzed with MO.Affinity Analysis v2.3 software (NanoTemper Technologies).

LutR was measured at a constant concentration of 8 μM. l-Lactate was diluted by 5 mM steps in the concentration range of 30–10 mM and further titrated by the two-fold serial dilution (10 mM–4.88 μM). Pyruvate was titrated by the two-fold serial dilution in the concentration range of 25 mM–3.05 μM. All samples were prepared in the LutR storage buffer [20 mM Tris·HCl, pH 7.5, 250 mM NaCl, 0.02% (v/v) β-mercaptoethanol]. LutR–l-lactate samples were measured in three independent measurements. The last measurement was performed three times under the same instrumental conditions and used for the data analysis.

### Crystallization of LutR–DBD

First, the suitability of the protein concentration for crystallization experiments was examined using a PCTTM Pre-Crystallization Test (Hampton Research, USA). The crystal used for X-ray measurement was obtained using the hanging-drop vapor-diffusion technique carried out in an EasyXtal 15-well plate (NeXtal Biotechnologies, Holland) at 18 °C. The crystal grew in the crystallization solution from the Morpheus crystallization screen (Molecular Dimensions, USA) consisting of a 0.1 M sodium HEPES/MOPS (acid) buffer system (pH 7.5), 37.5% (v/v) 2-methyl-2,4-pentanediol, 37.5% (v/v) PEG 1000, 37.5% (v/v) PEG 3350, 0.03 M NaF, 0.03 M NaBr, and 0.03 M NaI. This solution was mixed with the LutR–DBD in a volume ratio of 2 μl : 1 μl. The crystal was flash-cooled in liquid nitrogen and subjected to measurement.

### X-Ray data collection and data processing

The diffraction data were collected at 100 K at the BESSY II electron-storage ring in the Helmholtz-Zentrum Berlin, Germany.^49^ The data were processed using the XDSAPP software system.^50,51^ The data collection statistics are summarized in Table S1 (ESI†).^52–54^

The LutR–DBD structure was determined by molecular replacement with MOLREP^55^ from the CCP4 package^56^ using the highest-scored model generated by the I-TASSER server^57^ predicted based on the provided amino-acid sequence of the LutR–DBD (residues 2–78). The initial structure refinement was performed using the CCP4i program REFMAC5.8.0266,^58^ with the final stages carried out using the CCP4i2 program^59^ REFMAC5.8.0415.^58^ Computational refinement was combined with manual adjustments in COOT.^60,61^ The final model was evaluated with the help of the MOLPROBITY server.^62,63^ Upon validation, the diffraction data and the structure coordinates were deposited in the Protein Data Bank under accession code 8PQM. Structure visualizations were designed in the program PYMOL,^64^ and the secondary structure prediction and diagram were created using the ESPript 3.0 server.^65^

### Enzymatic synthesis and characterization of TBdp-modified DNA

Oligonucleotides were purchased from Generi Biotech (Czechia). Natural nucleoside triphosphates (dATP, dCTP, dGTP and TTP) were obtained from New England Biolabs. KOD XL DNA polymerase and the corresponding reaction buffer were purchased from Merck Millipore. Lambda exonuclease with the corresponding reaction buffer was obtained from New England Biolabs. The ultra-low ladder from Invitrogen® was purchased from Thermo Fisher. All solutions for biochemical reactions were prepared using Milli-Q water. Polyacrylamide and agarose gels were visualized using a fluorescence scanner (Typhoon FLA 9500) using cut-off filters when needed. Mass spectra of oligonucleotides were measured by MALDI-TOF mass spectrometry using an UltrafleXtreme MALDI-TOF/TOF instrument (Bruker Daltonics, Germany) with a 1-kHz Smartbeam II laser. The quantity of non-labelled oligonucleotides was measured using a spectrophotometer (Nanodrop 1000).

### Incorporation of fluorescent nucleotides by primer extension (PEX)

#### Protocol for single-strained DNA formation using lambda exonuclease digestion (protocol A)

The reaction mixture (50 μl) was prepared by mixing each purified oligonucleotide in the range of 1–10 μg or 10–25 μg, Lamda exonuclease buffer 10× (5 μl) and the lambda exonuclease enzyme (1 μl for quantities below 10 μg of oligonucleotide or 2 μl for quantities between 10 and 25 μg of oligonucleotide) at 37 °C for 1.5 h. The samples were purified using the QIAquick® Nucleotide Removal Kit obtained from Qiagen, following the supplier's instructions with a final elution step using 50 μl of UHPLC water.

#### Preparation of oligonucleotides containing one or two dC^TBdp^ modifications

The reaction mixture (120 μl) contained KOD XL DNA polymerase (1.8 μl, 2.5 U μl^−1^), KOD XL reaction buffer 10× (12 μl), prim^LutR1C^ or prim^LutR^ (100 μM, 6 μl), temp^5PLutR^ (100 μM, 6 μl) (see Table S2, ESI†), natural dNTPs (dATP, dGTP and TTP; 4 mM, 2.4 μl) and dC^TBdp^TP (4 mM, 4.8 μl). The samples were incubated for 1.5 h at 60 °C in a thermomixer, and the reactions were stopped by cooling to 4 °C. The samples were then purified using the QIAquick® Nucleotide Removal Kit from Qiagen following the supplier's instructions with a final elution step using 50 μl of UHPLC water. The obtained yields were 237 ng μl^−1^ of oligonucleotides with single dC^TBdp^ modification (1C^TBdp^) and 225 ng μl^−1^ of oligonucleotides with double dC^TBdp^ modification (2C^TBdp^). An aliquot of the samples was treated using protocol A.

#### Preparation of Cy5-labeled oligonucleotides containing one or two dC^TBdp^ modifications

Cy5-labeled oligonucleotides were prepared for the EMSA experiments. A fluorescent label was attached to the 5′ end of the primer. The reaction mixture (105.6 μl) contained KOD XL DNA polymerase (1.65 μl, 2.5 U μl^−1^), KOD XL reaction buffer 10× (11 μl), prim^LutR1C^-Cy5 or prim^LutR^-Cy5 (100 μM, 5.5 μl), temp^5PLutR^ (100 μM, 5.5 μl) (see Table S2, ESI†) and natural dNTPs (dATP, dGTP and TTP; 4 mM, 2.2 μl). The reaction mixture was divided into three aliquots. A positive control contained: dCTP (4 mM, 0.8 μl) and 19.2 μl of the reaction mixture; a negative control contained: UHPLC water 0.8 μl and 19.2 μl; experiments with singly or doubly modified dC^TBdp^ were carried out with dC^TBdp^TP (4 mM, 2.4 μl) and 57.6 μl of the reaction mixture, respectively. The samples were incubated for 1.5 h at 60 °C in a thermomixer, and the reactions were stopped by cooling to 4 °C. The samples were purified using the QIAquick® Nucleotide Removal Kit (Qiagen) following the supplier's instructions with a final elution step using 50 μl of UHPLC water. The obtained yields were 38 ng μl^−1^ of Cy5-labeled oligonucleotides in the case of the positive control, 38 ng μl^−1^ of Cy5-labeled oligonucleotides in the case of the negative control, 110 ng μl^−1^ of Cy5-labeled oligonucleotides in the case of the dC^TBdp^ singly modified oligonucleotide (1C^TBdp^) and 113 ng μl^−1^ in that of the Cy5-labeled dC^TBdp^ doubly modified oligonucleotide (2C^TBdp^).

### Interactions of 27DNA_2C^TBdp^ and LutR protein analyzed by an electrophoretic mobility shift assay (EMSA)

A series of solutions (each of a total volume of 6 μl) contained Cy5-27DNA_2C^TBdp^ (100 nmol; 1 μl), KCl (500 mM, 1 μl), VP buffer (50 mM Tris·HCl, 0.1% Triton-X100, pH 7.6, 2 μl), LutR storage buffer (250 mM NaCl, 20 mM Tris·HCl pH 7.5, 5 mM DTT; either 0, 1, 1.5, or 2 μl), and LutR stock solution (1.77 μg μl^−1^ in 250 mM NaCl, 20 mM Tris·HCl pH 7.5, 5 mM DTT; either 0, 0.5, 1, or 2 μl). Consecutively, the positive control was prepared under the same conditions as above but using Cy5-labeled DNA containing only natural nucleotides. To test the effect of l-lactate on DNA binding, a sample containing Cy5-27DNA_2C^TBdp^ (100 nmol; 1 μl), VP buffer (50 mM Tris·HCl, 0.1% Triton-X100, pH 7.6, 2 μl), LutR stock solution (1.77 μg μl^−1^ in 250 mM NaCl, 20 mM Tris·HCl pH 7.5, 5 mM DTT; 2 μl) and a solution of KCl: l-lactate (500 mM: 200 mM, 1 μl) was prepared. All solutions were incubated on ice for 30 min, then glycerol was added (80% in water; 1 μl). The reaction mixtures were run in a native 5% PAGE using a Tris–borate–EDTA buffer (TBE 0.5×) at 50 V for 3 h, keeping the low temperature (max 10 °C). The EMSA assay was visualized using a Typhoon fluorescent scanner with a 633-nm laser.

### Time-correlated single photon counting (TCSPC)

Experiments were performed in a 15-μl quartz cuvette. The temperature in the cuvette holder was maintained to 5 °C (±0.1 °C) using a water circulating bath. TCSPC was performed on a 5000U Single Photon Counting setup (IBH, UK) using a pulsed diode laser (470 nm, LDH-P-C-470, 75 ps pulse width, 5 MHz repetition rate, PicoQuant, Berlin, Germany) and a cooled Hamamatsu R3809U-50 microchannel plate photomultiplier. The emission wavelength selected using a monochromator was 515 ± 4 nm. Scattered light was further suppressed by a >500 nm cutoff filter. The signal level was kept below 2% of the light source repetition rate.

The time resolution, calculated as one-fifth of the full-width at half-maximum (FWHM) of the instrument's response function, was 100 ps. Mean fluorescence lifetime was calculated using the expression:
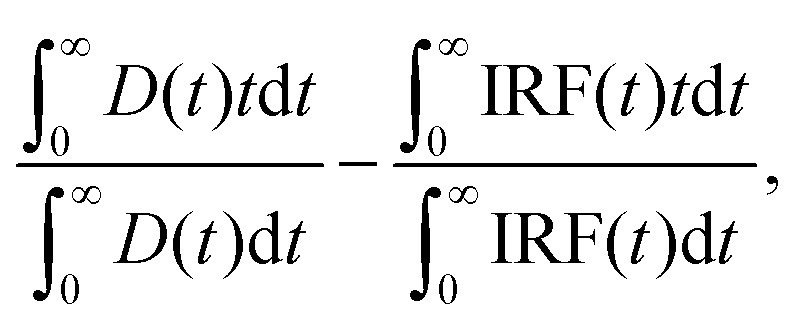
where “*D*(*t*)” is the measured decay, “IRF(*t*)” is the instrument response function measured for a scattering suspension of colloidal silica (Ludox, Sigma-Aldrich), and “*t*” stands for time after electronic excitation. This approach gives reliable mean fluorescence lifetimes independent of any artifacts of multiexponential fitting.

### Titration of TBdp-labeled DNA with LutR using TCSPC detection

A series of solutions (the total volume of each solution being 17 μl) contained 27DNA_1C^TBdp^ or 27DNA_2C^TBdp^ (0.32 μM, 10 μl), KCl (500 mM, 2 μl), VP buffer (50 mM Tris·HCl, 0.1% Triton-X100, pH 7.6, 2 μl), LutR storage buffer (250 mM NaCl, 20 mM Tris pH 7.5, 5 mM DTT; either 4, 3.5, 3, 2, or 0 μl) and LutR stock solution (2.5 μg μl^−1^ in 250 mM NaCl, 20 mM Tris·HCl pH 7.5, 5 mM DTT; either 0, 0.5, 1, 2, or 4 μl). The solutions were incubated on ice for 2–3 h. TCSPC spectra were measured at 5 °C. The measurements were triplicated.

As a negative control, BSA was used. A series of solutions (the total volume of each solution being 17 μl) contained 27DNA_1C^TBdp^ or 27DNA_2C^TBdp^ (0.32 μM, 10 μl), KCl (500 mM, 2 μl), VP buffer (50 mM Tris·HCl, 0.1% Triton-X100, pH 7.6, 2 μl), LutR storage buffer (250 mM NaCl, 20 mM Tris·HCl pH 7.5, 5 mM DTT; either 4, 3.5, 3, 2, or 0 μl) and BSA stock solution (5 μg μl^−1^ in 250 mM NaCl, 20 mM Tris·HCl pH 7.5, 5 mM DTT; either 0, 0.5, 1, 2, or 4 μl). The solutions were incubated on ice for 10 min. TCSPC spectra were measured at 5 °C.

### Study of potential effectors of LutR using 27DNA_2C^TBdp^

The reaction mixture (95 μl) contained 27DNA_2C^TBdp^ (12.8 μM, 10 μl), KCl (500 mM, 20 μl), VP buffer (50 mM Tris·HCl, 0.1% Triton-X100, pH 7.6, 40 μl), DTT (2 mM, 20 μl), and LutR stock solution (1.8 μg μl^−1^ in 250 mM NaCl, 20 mM Tris·HCl pH 7.5, 5 mM DTT; 5 μl). The reaction mixture was incubated for 1.5 h on ice. Effectors (l-lactate, sodium acetate, sodium pyruvate, acetyl coenzyme A, and l-alanine, 200 mM) were added separately and additively in aliquots (20 μl) until the desired molar equivalents were reached. TCSPC spectra were measured at 5 °C.

## Data availability

The data supporting this article have been included as part of the ESI.† Crystallographic data for the structure of the LutR DNA-binding domain (residues 2–78) have been deposited at the Protein Data Bank (PDB) under accession code 8PQM.

## Conflicts of interest

There are no conflicts to declare.

## Supplementary Material

CB-OLF-D4CB00260A-s001
