# Supplementary material for: Using environment-sensitive tetramethylated thiophene-BODIPY fluorophores in DNA probes for studying effector-induced conformational changes of protein–DNA complexes
Source: RSC Chem Biol. 2025 Jan 2;6(3):376–86. doi: 10.1039/d4cb00260a (PMC11734750; doi:10.1039/d4cb00260a)
Supplement: CB-006-D4CB00260A-s001 [file CB-006-D4CB00260A-s001.pdf]

Supplementary Information (SI) for RSC Chemical Biology.
This journal is © The Royal Society of Chemistry 2024

Supplementary Information

Using environment-sensitive tetramethylated thiophene-BODIPY fluorophore in DNA probes for studying effector-induced conformational changes of protein-DNA complexes

Markéta Šoltysová[a#], Pedro Güixens-Gallardo[a#], Irena Sieglová[a], Anna Soldánová[a], Veronika Krejčiříková[a], Milan Fábry[a], Jiří Brynda[a], Petro Khoroshyy[a], Michal Hocek[a,b *], Pavlína Řezáčová[a,*]

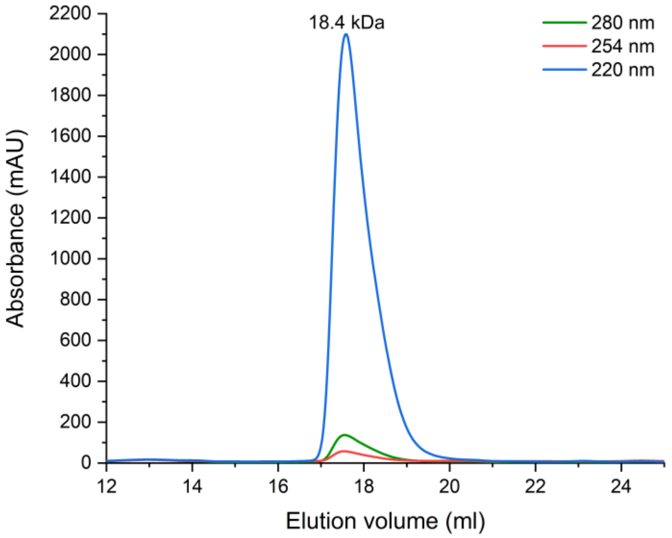

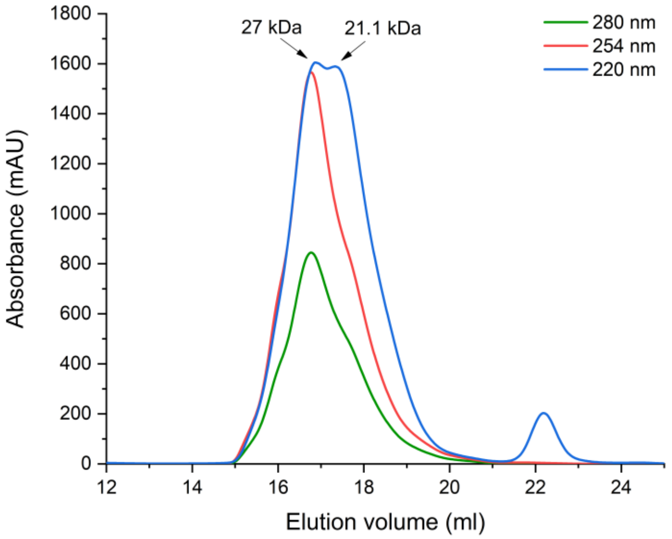

Figure S1: Analytical size-exclusion chromatography of (A) LutR-DBD (33.2 µM) and (B) LutR-DBD in the complex with a 15 bp long DNA operator (16.6 µM) variant. The apparent molecular weights of eluted components are indicated in the plots and were calculated based on the calibration equation $\log MW = -0.2362x + 8.4183$.

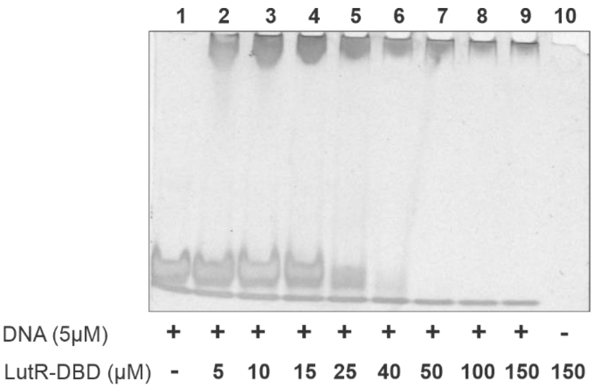

Figure S2. Electromobility shift assay of LutR-DBD (residues 2 – 78) and 15 bp DNA containing the inverted repeat (5′-GGTCATCAGATGACC-3′) in the 15% (w/v) polyacrylamide gel.

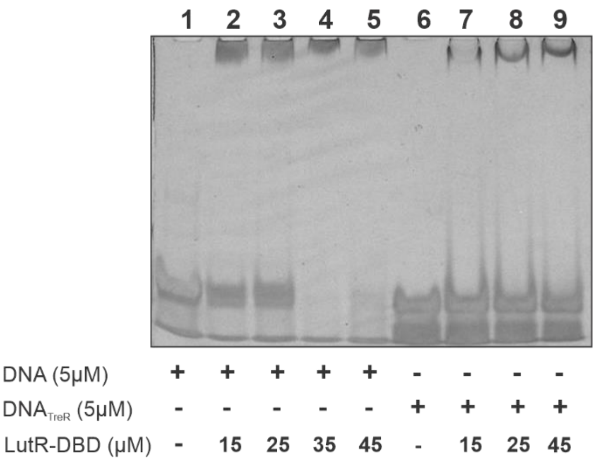

Figure S3. Electromobility shift assay of LutR-DBD (residues 2 – 78) and 15 bp DNA (DNA) containing the inverted repeat (5′-GGTCATCAGATGACC-3′) and an unrelated DNA operator of another protein from the GntR family, TreR, used as a negative control. The assay was run in the 17% (w/v) polyacrylamide gel.

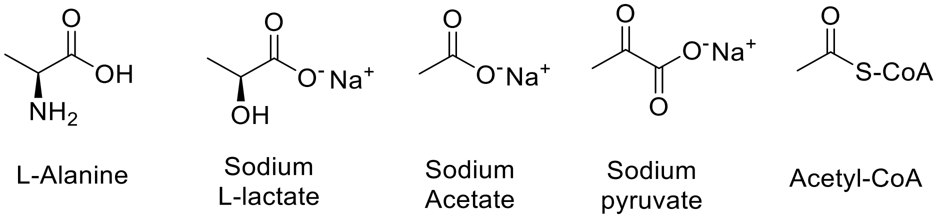

Figure S4. Formulas of metabolites tested for their effector activity.

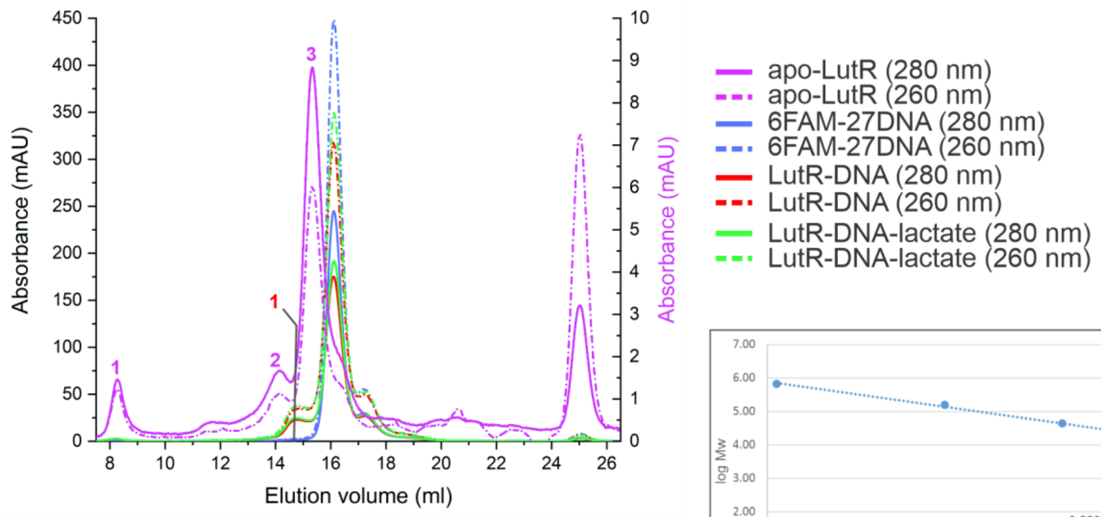

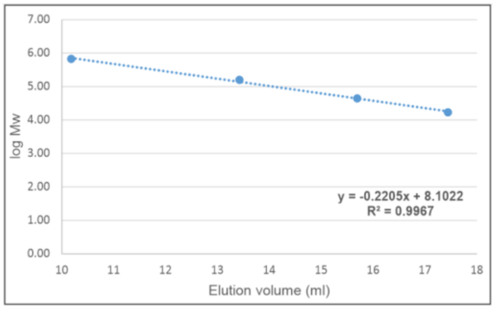

Sample	apo-LutR			LutR-DNA
Peak	1	2	3	1
Theoretical MW_{theor}	29.59 kDa/59.18 kDa (monomer/dimer)			75.74 kDa (dimer+DNA)
Elution volume	8.27 ml	14.12 ml	15.33 ml	14.81 ml
Apparent MW_{app} [a]	1,900 kDa	97.44 kDa	52.72 kDa	68.64 kDa
Assembly[b]	aggregate	trimer (3.3)	dimer (0.9)	dimer+DNA (0.9)

[a] $MW_{app} = 10^{(-0.2205 \times Ve + 8.1022)}$

[b] Assembly = MW_{app}/MW_{theor}

Figure S5. Size-exclusion chromatography analysis of LutR (pink), single 5'-6FAM labelled 27DNA (blue), and the mixtures of LutR-DNA (red) and LutR-DNA-L-lactate (green). Overlays of chromatograms at 280 and 260 nm are shown. For the low signal of the LutR apo-protein with respect to DNA and the mixtures, the second y axis (pink) was added. Elution volumes and calculations of the apparent molecular weights (MW_{app}) and assemblies of all the apo-LutR's peaks and the most relevant peak of the mixtures are indicated in the table. The calibration of the Superdex 200 10/300 GL column is presented on the right.

Table S1: Crystal data and diffraction data collection and refinement statistics

	**LutR-DBD**
**Data collection statistics**	
Space group	*P*222
Cell parameters (Å; °)	28.31, 38.37, 66.71; 90, 90, 90
Wavelength (Å)	0.9797
Resolution (Å)	50.0–1.46 (1.55–1.46) [a]
Number of unique reflections	24387 (3958)
Multiplicity	6.76 (6.55)
Completeness (%)	100.0 (100.0)
R_{meas} [b]	7.3 (338.8)
$CC_{(1/2)}$ [c]	99.9 (31.9)
Average I/δ(I)	12.26 (0.49)
Wilson B ($Å^2$)	33.47
**Refinement statistics**	
Resolution range (Å)	33.38–1.46
No. of reflections in working set	13,187
No. of reflections in test set	660
R value [d]	0.20
R_{free} value [e]	0.24
RMSD bond length (Å)	0.0093
RMSD angle (°)	1.572
Number of atoms in AU	
Protein	639
Water	81
Sodium ions	2
Mean B value ($Å^2$)	43.25
Ramachandran plot statistics	
Residues in favored regions (%)	96.0
Residues in allowed regions (%)	4.00
PDB code	8PQM

[a] The data in parentheses refer to the highest-resolution shell.
[b] R_{meas} – redundancy-independent R factor [63].
[c] $CC_{(1/2)}$ is the correlation coefficient between random half-datasets; from its value the Pearson correlation coefficient of the true level of signal can be calculated as follows: $CC = \sqrt{2\,CC_{1/2}/1 + CC_{1/2}}$. [64]
[d] R value = $||F_o| - |F_c|| / |F_o|$, where F_o and F_c are the observed and calculated structure factors, respectively.
[e] R_{free} is equivalent to the R value but is calculated for 5% of reflections chosen at random and omitted from the refinement process [65].
[f] as determined by Molprobity [59,60].

Table S2. List of oligonucleotides used

Oligonucleotide	Sequence 5′→ 3′ (a)	Length
prim^{LutR1C}	CAGAATAATGGGTCAGTAGTC	21-mer
prim^{LutR1C}-Cy5 (b)	CAGAATAATGGGTCAGTAGTC	21-mer
prim^{LutR}	CAGAATAATGGGTC	14-mer
prim^{LutR}-Cy5 (b)	CAGAATAATGGGTC	14-mer
temp^{5PLutR} (c)	CTAGTAGACTACTGACCCATTATTCTG	27-mer

[a] Primer sequences in the template are underlined.
[b] Cyanine-5 (Cy5) used for oligonucleotide labeling at the 5′ end.
[c] Template phosphorylated at the 5′ end

Table S3. Mean fluorescence lifetime of 27 bp long DNA (300 nM) bearing one (1C^{TBdp}) or two (2C^{TBdp}) modifications and titrated with LutR or BSA.

	Protein concentration (μM)	Mean lifetime of 27DNA_1C^{TBdp}		Mean lifetime of 27DNA_2C^{TBdp}	
		τ (ns)	±SD	τ (ns)	±SD
LutR	0	2.43	0.04	2.28	0.08
	1.5	3.9	0.05	3.38	0.20
	3.0	4.11	0.05	4.63	0.08
	6.0	4.33	0.11	4.90	0.02
	12.0	4.53	0.07	4.92	0.03
LutR + effector*	12.0	2.71	0.18	3.14	0.19
BSA	1.5	2.64	–	2.35	–
	3.0	2.59	–	2.54	–
	6.0	2.92	–	2.80	–
	12.0	3.04	–	2.71	–

*Sodium L-lactate was added to give a final concentration of 2.5 mM.
